# RNA methylation into m^1^A era: a new regulation over T-cell function

**DOI:** 10.1038/s41392-023-01360-4

**Published:** 2023-02-22

**Authors:** Ping Lin, Guoping Li, Min Wu

**Affiliations:** 1 Integrative Science Center of Germplasm Creation in Western China (Chongqing) Science City & Southwest University, Biological Science Research Center, Southwest University, 400715 Chongqing, China; 2 Wound Trauma Medical Center, State Key Laboratory of Trauma, Burns and Combined Injury, Daping Hospital, Army Medical University, 400042 Chongqing, China; 3 Wenzhou Institute, University of Chinese Academy of Sciences, Wenzhou, Zhejiang 325000 China; 4 Laboratory of Allergy and Precision Medicine, Chengdu Institute of Respiratory Health, The Third People’s Hospital of Chengdu, 610014 Chengdu, Sichuan China

**Keywords:** Non-coding RNAs, Biochemistry

In a recent paper published in *Nature Immunology*, Liu et al. described that the “writers” TRMT6/TRMT61A complex mediated transfer RNA (tRNA) m^1^A modification, facilitating competent translation of certain proteins fundamental to T-cell proliferation in an instant upon activation,^1^ demonstrating that tRNA m^1^A methylation as a crucial translational checkpoint paves the way for novel immunotherapies to treat T-cell-related inflammation or cancer via manipulating the m^1^A machinery.

**Fig. 1:**
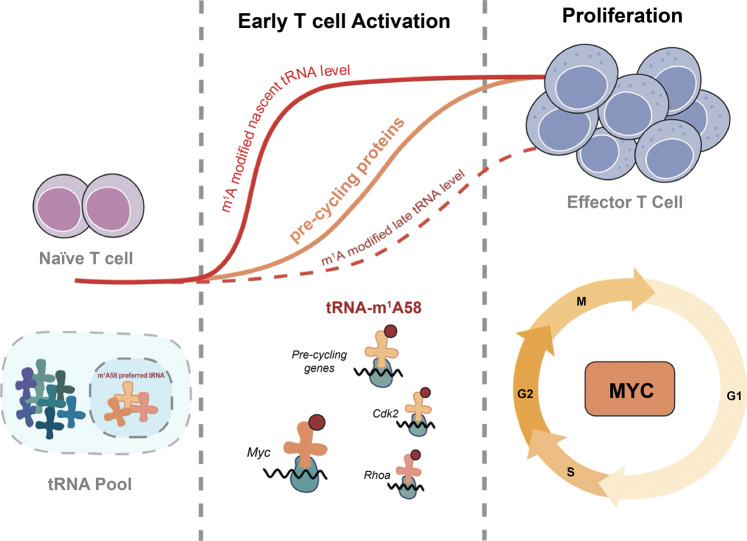
TRMT61A-mediated tRNA-m^1^A58 modification serves as a novel “translation checkpoint” for CD4^+^ T-cell proliferation. In the stage of early T-cell activation, efficient translation control is activated by TRMT61A/TRMT6-mediated tRNA-m^1^A58 modification on a subset of early upregulated tRNA to enable synthesis of MYC and of a specific group of key functional proteins, which is required to promote rapid T cell into cell cycles and expansion

As part of adaptive immunity, CD4^+^ T cells play an instrumental role. In order to provide adequate immune defense, naïve T cells require the timely synthesis of a large number of functional proteins upon antigen stimulation so as to accommodate the drastic increase in bioenergetic and biosynthetic demands necessary to exit quiescent state and undergo massive clonal expansion and differentiation (Fig. 1).^1^ Researches in the past few years have led to significant progress toward understanding the mechanism of T-cell activation upon antigen stimulation,^2^ which has been extensively learned on the transcriptional level. However, little is known about how other phases of protein translation, especially tRNA-mediated translation, affect T-cell responses.

It has long been believed that tRNAs influence translation through their interactions with the codons on mRNA. Recently, a translation control model based on *N*^1^-methyladenosine modification (m^1^A) of tRNAs explains the protein synthesis regulation. m^1^A modification in tRNA produced by “writers” TRMT6/TRMT61A complexes allow efficient translation elongation of mRNAs. Based on this model, as well as the dilemma of massive protein synthesis demand during T-cell activation, Liu et al. hypothesized that tRNA-m^1^A modification may contribute to accelerated mRNA translation efficiency and protein synthesis, thus ensuring rapid T-cell proliferation. To this end, they performed time point RNA-sequencing and tRNA-sequencing at four different stages of activation, namely early signaling activation, metabolic reprogramming, pre-cell-cycling, and proliferation. The results demonstrated that early T-cell activation was dominated by translation events, and the expressions of most tRNAs and tRNA processing-related genes were greatly upregulated. Furthermore, they found that Trmt61a and Trmt6 were rapidly upregulated upon T-cell activation.

Under physiological conditions, the increase of m^1^A writers may have two effects: one is to cause a proportional increase in the modification within the tRNA pool, and the other is to maintain the modification at the same proportions as seen on the tRNA in naïve cells as global tRNA levels increase. Benefitted from the recent progress on the m^1^A detection technology,^3^ the authors performed tRNA methylation sequencing to detect the tRNA-m^1^A levels in T cells, and found that general tRNA m^1^A level was steady during T-cell activation, implying that TRMT6/TRMT61A complex is scaled up to maintain the same proportions of m^1^A tRNA modification levels upon T-cell activation. After a series of in vitro and in vivo tests, the authors proved that tRNA-m^1^A58 prompted the rapid proliferation of T cells and timely immune responses by regulating the translation elongation of certain cell cycling mRNAs (Fig. 1). *Trmt61a* depletion results in a significant reduction in the m^1^A58 modification level of most tRNAs in CD4^+^ T cells and a significant decrease in translational decoding ability, ultimately blocking the translation of numerous key proteins, particularly MYC, a transcription factor that must be expressed rapidly during CD4^+^ T-cell activation. The reduced protein level of MYC results in disruption of metabolic reprogramming and cell cycle, ultimately preventing CD4^+^ T-cell clonal expansion.

Further questions that need to be addressed were how TRMT61A affects gene translation and what properties and functions these TRMT61A-specific genes have. Based on the idea that the genetic information decoding process is directly affected by the size and composition of the tRNA pool, Liu et al. examined gene-specific preferential translation biases from three perspectives: tRNA expression dynamics, codon utilization of various mRNAs, and tRNA sensitivity to TRMT61A deletions. Six clusters of tRNAs were identified based on their expression profiles. T1 and T2 tRNA clusters were significantly increased in the early phase of T-cell activation, whereas the other four tRNA clusters showed a minor change in expression levels over time. In general, different tRNAs respond differently to TRMT61A deletion, and m^1^A58 modification of the T1 and T2 cluster tRNAs is significantly impacted by TRMT61A expression. In addition, mRNAs with decreased translation efficiency that corresponds to T1 and T2 cluster tRNAs have significantly higher codon usage rates than mRNAs with increased or unchanged translation efficiency. Collectively, the translation bias is caused by the different utilization rates of the most rapidly induced tRNAs with the highest level of m^1^A modification and high sensitivity to TRMT61A deletion. Aside from leading to a deeper understanding of RNA epigenetics in immunology, this discovery opened up a broad range of possibilities for investigating the role of m^1^A in human disease and health.

The role of tRNAs, tRNA modifications, and selective codon usage is almost completely unexplored in immunology. Liu and colleagues present the first mechanistic evidence linking tRNA function changes to T-cell tRNA modification in this exciting study. It demonstrates RNA-m^1^A modification to be a new translational checkpoint that stimulates T-cell proliferation, uncovering a new layer of post-transcriptional regulation that plays a role in T-cell homeostasis and immune response. Another recent research also showed that m^1^A modification was increased in a subset of tRNAs in liver cancer to promote cholesterol metabolism and drive liver cancer stem cell renewal and tumourigenesis.^4^ These studies in m^1^A RNA methylation reveal the importance of this new type of RNA methylation, other than the well-known m^6^A methylation, and pave the way for novel immunotherapies to treat T-cell-related inflammation or cancer via manipulating the m^1^A machinery. Moreover, there are additional sites in tRNAs subject to m^1^A methylation modification by other types of “m^1^A writers”, as well as dozens of other types of epigenetic modifications such as pseudouridylation.^5^ It will be extremely interesting to explore in the future how those tRNA modifications regulate translation and T-cell function, and how those different methylations cross-talk to coordinately respond to external stimuli. We are into a modified RNA world.
